# Association between socioeconomic factors and origin of hospital referrals among patients with oral cancer

**DOI:** 10.4317/medoral.25478

**Published:** 2022-06-19

**Authors:** Maria Letícia B Raymundo, Leonardo de Freitas Ferreira, Deborah E W Gomes-Freire, Aldelany R Freire, Rennis O Silva, Elza C F Araújo, Edson H G Lucena, Yuri W Cavalcanti

**Affiliations:** 1 ORCID: 0000-0002-5560-2223. School of Dentistry. Federal University of Paraíba (UFPB), João Pessoa, Brazil; 2 ORCID: 0000-0002-4948-4347. School of Dentistry. Federal University of Paraíba (UFPB), João Pessoa, Brazil; 3 ORCID: 0000-0002-0001-7430. Post-graduation Program in Dentistry (PPD) of the Federal Univ. of Paraíba (UFPB), João Pessoa, Brazil; 4 ORCID: 0000-0002-8082-5883. Post-graduation Program in Dentistry (PPD) of the Federal Univ. of Paraíba (UFPB), João Pessoa, Brazil; 5 ORCID: 0000-0001-8413-8071. Post-graduation Program in Dentistry (PPD) of the Federal Univ. of Paraíba (UFPB), João Pessoa, Brazil; 6 ORCID: 0000-0002-8303-8914. Post-graduation Program in Dentistry (PPD) of the Federal Univ. of Paraíba (UFPB), João Pessoa, Brazil; 7 ORCID: 0000-0003-3431-115X. Department of Clinical and Social Dentistry. Federal Univ. of Paraíba (UFPB), João Pessoa, Brazil; 8 ORCID: 0000-0002-3570-9904. Department of Clinical and Social Dentistry. Federal Univ. of Paraíba (UFPB), João Pessoa, Brazil

## Abstract

**Background:**

The Brazilian Unified Health System (SUS) is responsible for offering free assistance to more than 100 million Brazilians, including treatment of oral cancer lesions. Considering that the Brazilian public system aids the most vulnerable population, this study analyzed whether the origin of hospital referrals of patients with oral cancer is associated with socioeconomic factors.

**Material and Methods:**

A cross-sectional study was carried out from cancer hospital records of the National Cancer Institute (RHC-INCA), considering the primary locations (C00 to C06) diagnosed between 2016 and 2019. Data on gender, skin color (white and non-white), education (no schooling, incomplete or complete elementary education; high school; incomplete and complete higher education) and origin of referral (SUS and non-SUS) were analyzed by multiple logistic regression (*p*<0.05).

**Results:**

Higher referral rates by the SUS were observed in 2017 (OR=1.27; 95% CI=1.098-1.480) and 2018 (OR=1.28; 95% CI=1.101-1.490); no differences were found between the years 2016 and 2019. Regarding gender, men were 40% more likely to have the SUS as the source of referral (OR=1.40; 95% CI=1.233-1.600). Non-white individuals were 34% more likely to have the SUS as the source of the referral (OR=1.34; 95% CI=1.190-1.512). Illiterate individuals or individuals who only attended elementary school were 6.38 times more likely to be referred by the SUS than individuals with higher education (OR=6.38; 95% CI=5.228-7.796).

**Conclusions:**

It is concluded that the origin of hospital referrals via SUS of patients with oral cancer is associated with socioeconomic factors.

** Key words:**Mouth neoplasms, oral diagnosis, health service coverage.

## Introduction

Oral cancer involving the lip and oral cavity regions stands out among head and neck tumors due to its high incidence, morbidity, and mortality (1). The highest incidence and mortality rates of lip and oral cavity cancers are observed for men, and an increasing incidence are also observed for women, within the 1990 and 2019 period (2).

The epidemiological profile of the oral cancer disease consists of men, aged between 40 and 60 years (2), mostly with low socioeconomic status and low education (3). The main risk factors are smoking and alcohol abuse (4).

The treatment of choice for tumors of the lip and oral cavity in early stages is surgery. However, radiotherapy and chemotherapy are used combined with surgery, or alternatively, in cases where surgery is not indicated (5,6).

As of 2004, the National Oral Health Policy (PNSB) allowed the expansion of the Primary Care coverage through the Family Health Strategy (ESF), as well as expanding Secondary Care, through Dental Specialty Centers (CEO) (7). Educational actions to combat risk factors for oral cancer and early detection of changes in the oral mucosa are prioritized in primary care. In general, suspected cases of oral cancer identified in primary care are referred to specialized care, represented by the CEO, where most biopsies are performed (8). The oncological treatment of the most severe cases is carried out in reference hospital units (9).

A better prognosis can be achieved when the diagnosis is made early (10). However, the high rate of morbidity and mortality from oral cancer in Brazil is due to late hospitalization of patients in advanced stages of the disease (1,3,5).

Late diagnosis often occurs due to lack of access to health services (11). Population in situation of social vulnerability are the most affected by the lack of access. However, a large part of the Brazilian population depends on health care provided by the Unified Health System (SUS) (12). Although there are several challenges in the diagnosis and prevention of oral cancer, the increase in health coverage and the increase in the number of Dental Specialty Centers contributed to a reduction in the number of hospitalizations due to oral cancer in Brazil (4).

In Brazil, hospitals are required to notify cancer cases that progress to hospital admissions. These cases are registered through the Hospital Cancer Records Integrator linked to the José de Alencar Gomes da Silva National Cancer Institute (INCA). These records allow to monitoring the severity of cancer cases, as well as their possible relationship with socioeconomic factors (12). Some studies from our research group have used the Hospital Cancer Records from INCA to discuss the association between oral cancer, socioeconomic factors and public health coverage (3,4,12).

The records of oral cancer cases that require hospitalization allow monitoring the care provided to the patient. It is not known, however, whether the assistance provided in the Unified Health System is in line with the principle of equity, in which assistance should preferably be offered to the most vulnerable groups.

Therefore, this study aimed to verify whether the origin of hospital referrals of patients with oral cancer is associated with socioeconomic factors.

## Material and Methods

- Study design

An observational, cross-sectional study was carried out to verify the origin of hospital referrals for patients with oral cancer and its association with socioeconomic factors.

- Data collection

Data were obtained from the Hospital Cancer Records of the National Cancer Institute (RHC – INCA) (https://irhc.inca.gov.br/RHCNet/visualizaTabNetExterno.action). Data were collected according to the origin of the hospital referral (SUS and non-SUS) considering the primary locations: lip, base of the tongue, tongue, gum, floor of the mouth, palate, other unspecified parts of the mouth (C00 to 06).

In addition, more detailed information was collected for each oral cancer lesion in the RHC-INCA database, such as gender, skin color (white and non-white), education (no schooling, incomplete or complete elementary education; high school; incomplete and complete higher education) and year of referral, considering the period from 2016 to 2019.

- Data analysis 

The frequencies of all individual variables were obtained to characterize the sample. A multiple logistic regression was generated between the independent variables (gender, skin color, education, and year of hospital referral) and the outcome variable (origin of hospital referral), considering a value of *p*<0.05 and a confidence interval of 95% as significant. All analyses were performed using the Jamovi® software.

## Results

Of the analyzed sample, 75.4% are men. Regarding skin color, 58.2% of the individuals declared themselves to be non-white (black, brown, yellow, and indigenous). Regarding the education variable, 82.9% of individuals have no schooling or have elementary education (complete or incomplete). As for the origin of the hospital referral, 88.5% were referred via SUS (Table 1).

**Table 1:**
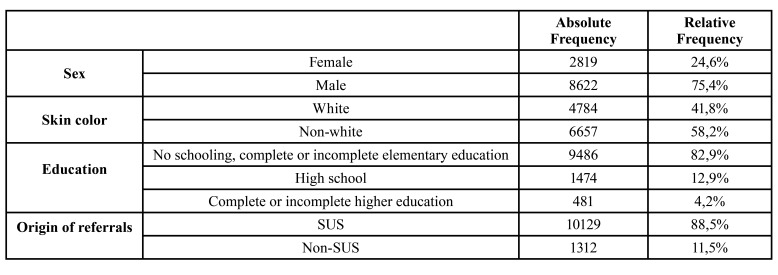
Descriptive statistics of the variables that were studied.

Higher referral rates by the SUS were observed in 2017 (OR=1.27; 95% CI=1.098-1.480) and 2018 (OR=1.28; 95% CI=1.101-1.490); no differences were found between the years 2016 and 2019. Regarding the gender variable, males were 40% more likely to have the SUS as the source of referral (OR=1.40; 95% CI=1.233-1.600). Regarding skin color, non-white individuals were 34% more likely to have the SUS as the source of referral (OR=1.34; 95% CI=1.190-1.512). Illiterate individuals or individuals who attended elementary school were 6.38 times more likely to be referred by the SUS than individuals with higher education (OR=6.38; 95% CI=5.228-7.796), and individuals who attended high school were 2.13 times more likely to have the SUS as the origin of the referral than individuals with higher education (OR=2.13; 95% CI=1.706-2.67) (Table 2).

**Table 2:**
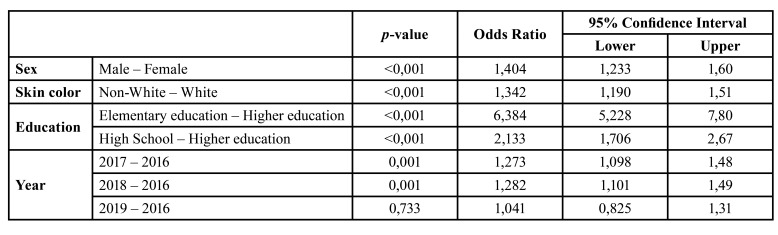
Logistic regression between origin of treatment and socioeconomic changes.

## Discussion

The results of the present study indicate that the profile of individuals referred for diagnosis and treatment of oral cancer is compatible with the epidemiological profile of Brazil and with the profile of users assisted by the SUS. According to the results of this study, non-white male individuals with no education or only elementary education are referred and assisted by the SUS when diagnosed with oral cancer.

The skin color variable is an important marker of inequity in access to health care. Black individuals have a worse prognosis for oral cancer due to late diagnosis of the disease, or difficult access to health care (11,13). However, the SUS plays an important role in reducing these inequities by prioritizing the most vulnerable populations, to improve the epidemiological picture of oral cancer in Brazil (12). According to the results of the present study, non-white individuals were the majority in the SUS referral to the hospital service.

In addition to skin color being associated with situations of vulnerability and low level of education (13), socially underprivileged groups tend to have greater contact with tobacco and alcoholic beverages, which are risk factors for the development and worsening of cancerous injuries. (4,14).

A lower level of education is strongly associated with the risk of developing oral cancer, and is associated with worse clinical outcomes, including death (4,15). Individuals with low income and low education have less access to health services (13), and according to the results of the present study, hospital referrals carried out by the SUS guarantee assistance to less privileged populations, seeking to reduce health inequities.

The PNSB reorganized oral health care, expanding access through the expansion of primary health care (PHC) and ensuring continuity of care at a specialized level (16). However, some PHC dentists consider themselves unprepared to perform a biopsy, a necessary procedure for diagnosis and treatment (17). The referral for the procedure can delay the diagnosis and result in a not-so-favorable prognosis (11). However, the data from this study shows that about 90% of cancer cases registered at INCA had the SUS as the origin of the referral, showing that the SUS is responsible for aiding in most cases registered in the RHC of the INCA.

The referral of individuals to the hospital level, starting from the basic health care units and passing through, or not, specialized care, represents the integrality of care. The present study shows that the origin of hospital referral is associated with socioeconomic variables, making it evident that the SUS contributes to the reduction of health inequities, respecting the principle of equity. Identifying these inequities regarding the profile of referred users, where the most socioeconomically vulnerable receive assistance, shows the positive effects of the PNSB and the expansion of primary and secondary care in oral health.

## Conclusions

The origin of hospital referrals via SUS of patients with oral cancer is associated with socioeconomic factors. Male, self-declared non-white individuals, with low education are the majority in hospital referrals by the SUS, emphasizing the role of the SUS in reducing inequities and access to health.
